# In Vitro Comparison of the *Acanthamoeba* Cysticidal Activity of Povidone Iodine, Natamycin, and Chlorhexidine

**DOI:** 10.1016/j.xops.2021.100025

**Published:** 2021-05-03

**Authors:** Travis K. Redd, Maya Talbott, Vicky Cevallos, Prajna Lalitha, Gerami D. Seitzman, Thomas M. Lietman, Jeremy D. Keenan

**Affiliations:** 1 Francis I. Proctor Foundation, University of California, San Francisco; 2 Casey Eye Institute, Oregon Health & Science University, Portland, Oregon; 3 Aravind Eye Hospital, Madurai, India; 4 Department of Ophthalmology, University of California, San Francisco

**Keywords:** Acanthamoeba, Chlorhexidine, Iodine, Natamycin, IQR, interquartile range, MCC, minimum cysticidal concentration

## Abstract

**Purpose:**

*Acanthamoeba* keratitis often is refractory to medical and surgical therapy, primarily because of the remarkable resilience of *Acanthamoeba* cysts. In this study, we directly compared the cysticidal activity and potency of several candidate medical therapies in vitro.

**Design:**

Experimental study.

**Participants:**

In vitro *Acanthamoeba* specimens obtained from 9 patients with keratitis seen at the Francis I. Proctor Foundation from 2008 through 2012.

**Methods:**

The minimum cysticidal concentration (MCC) of povidone iodine, natamycin, and chlorhexidine was investigated using an established assay technique. The relative potency of each agent was estimated starting with concentrations commonly used in clinical practice and determining the number of two-fold dilutions required to reach the MCC. Statistical comparisons of relative potency were performed using bootstrap simulations and permutation tests.

**Main Outcome Measures:**

Minimum cysticidal concentration and the number of two-fold dilutions required to reach the MCC.

**Results:**

The MCC for chlorhexidine ranged from 3.1 to 25 μg/ml (median, 12.5 μg/ml; interquartile range [IQR], 6.25–12.5 μg/ml), the MCC for natamycin ranged from 390.6 to 3125 μg/ml (median, 390.6 μg/ml; IQR, 390.6–781.2 μg/ml), and the MCC for povidone iodine ranged from 0.3 to 78.1 μg/ml (median, 2.4 μg/ml; IQR, 0.6–9.8 μg/ml). Doses commonly used in clinical practice (povidone iodine 1%, natamycin 5%, and chlorhexidine 0.04%) were approximately 12, 7, and 5 two-fold dilutions higher than the drug’s corresponding median MCC, respectively (*P* < 0.001, comparing 3 drugs). Povidone iodine 1% had the highest potency of the 3 medications tested, requiring more dilutions than natamycin 5% (*P* < 0.001) and chlorhexidine 0.04% (*P* < 0.001) to reach the MCC.

**Conclusions:**

All 3 medications demonstrated in vitro cysticidal activity in each of the 9 isolates. The potency of 1% povidone iodine was greater than standard formulations of natamycin or chlorhexidine. Although its clinical efficacy is yet to be determined, povidone iodine may be considered as a potential adjuvant treatment in cases of recalcitrant *Acanthamoeba* keratitis.

*Acanthamoeba* is a genus of free-living, cyst-forming protozoa that are ubiquitous in soil and water. Most humans have been exposed to and carry immunoglobulin G antibodies to *Acanthamoeba* species without incident, but rarely, they can cause serious infection, including encephalitis and keratitis.^1^ The incidence of *Acanthamoeba* keratitis in the United States and United Kingdom has increased steadily in recent years, particularly among contact lens wearers.^2^, ^3^, ^4^ The life cycle of amoebae consists of a metabolically active trophozoite phase and a dormant cyst phase, both of which typically are present in the setting of *Acanthamoeba* keratitis. Trophozoites are eradicated relatively easily with medical therapy, whereas cysts are remarkably resistant to extreme temperatures, noxious stimuli, and chemical agents. Effective management of *Acanthamoeba* keratitis requires eradication of both trophozoites and cysts.^5^, ^6^, ^7^, ^8^

Medical therapy is the mainstay of *Acanthamoeba* keratitis management, but significant variability exists in the medication regimens used, largely because of limited clinical evidence to guide treatment.^9^ First-line therapy long has consisted of topical biguanide antiseptics and diamidine agents, although other medications including azole antifungals sometimes are added.^6^^,^^10^^,^^11^ In vitro testing of the cysticidal activity of a variety of other antimicrobial agents, including povidone iodine and natamycin, has demonstrated mixed results.^12^, ^13^, ^14^, ^15^, ^16^, ^17^, ^18^ Interpretation of these findings is limited by the wide variability in cysticidal assay methodology, few direct comparisons between antimicrobial agents, and limited evaluation of the relationship between the medication concentration required to achieve cysticidal effect in vitro and the concentrations typically used in clinical practice. In this study, we evaluated the in vitro cysticidal activity and directly compared the potency of povidone iodine, natamycin, and chlorhexidine using a well-established *Acanthamoeba* cysticidal assay technique.

## Methods

### *Acanthamoeba* Isolates

Nine *Acanthamoeba* isolates were obtained from corneal scrapings of patients with infectious keratitis at the Francis I. Proctor Foundation between 2008 and 2012. Scrapings originally were plated on nonnutrient agar with 0.5 McFarland standard *Escherichia coli* overlay and incubated at 30° C. *Acanthamoeba* organisms that grew from the clinical scraping were left in their original media and allowed to encyst spontaneously (i.e., the so-called time method of encystment).^19^ Cysts subsequently were stored in the original petri dish at ambient temperature without addition of culture medium or bacteria. Before the cysticidal assay, a sample of cysts was obtained from the storage petri dish and replated on nonnutrient agar with *E. coli* overlay to induce excystment. Cysts subsequently were allowed to encyst via the time method described above, and then transferred to normal saline and titrated to a concentration of 10^4^ cysts/ml with the assistance of a hemocytometer.

### Antimicrobial Microdilution

Stock solutions consisted of chlorhexidine 0.04% (400 μg/ml; Leiter’s Pharmacy), povidone iodine 1% (10 000 μg/ml; Alcon), and natamycin 5% (50 000 μg/ml; Alcon). Using sterile water, 8 two-fold dilutions of the chlorhexidine and natamycin stock solutions were made and 16 two-fold dilutions of the povidone iodine stock solution were made; the additional dilutions were needed for povidone iodine because of nearly universal cysticidal activity in the initial 8 dilutions. All dilutions were stored at 4° C.

### Cysticidal Assay

Cysticidal activity assays were performed in the Spring of 2015 using a previously described microdilution assay to establish the threshold cysticidal concentration for each of the 3 antimicrobial agents.^16^^,^^17^^,^^20^ This technique takes advantage of the fact that *Acanthamoeba* cysts adhere to the walls of a polycarbonate microtiter plate and remain attached even after drugs are added and removed. To set up the assay, 50-μl aliquots of increasingly concentrated serial antimicrobial dilutions were added to consecutive wells of a 96-well plate, with 8 wells used per assay for chlorhexidine and natamycin and 16 wells used per povidone iodine assay. Fifty-microliter aliquots of the previously prepared *Acanthamoeba* suspensions subsequently were added to each well, with a dedicated plate for each unique *Acanthamoeba* isolate. Two rows per plate were reserved for positive controls (i.e., *Acanthamoeba* suspension without drug) and negative controls (i.e., drug without *Acanthamoeba*). Each plate then was spun down at 1500 rpm for 5 minutes at room temperature, followed by incubation at 30° C for 48 hours. All plates were spun once more for an additional 5 minutes at 1500 rpm, and the fluid from each cell was aspirated carefully with a glass pipette and discarded. Each cell was washed with 100 μl of sterile Page saline and the solution was aspirated and discarded, with the entire process repeated for a total of 3 washings. The resulting samples then were incubated with an additional 100 μl of 0.5 McFarland standard *E. coli* suspension per cell at 30° C for 1 week to promote excystment and transition into the trophozoite form of any remaining viable cysts after this incubation period. On day 7, the entirety of each well was examined systematically to determine the presence (i.e., growth) or absence (i.e., no growth) of trophozoites using an inverted microscope at ×20 magnification. The minimum cysticidal concentration (MCC) of each medication for a given *Acanthamoeba* specimen was designated as the lowest drug concentration at which no trophozoites were evident on examination at day 7 of incubation. All plates demonstrated growth in the positive control wells and no growth in the negative control wells.

### Statistical Analysis

The primary outcome of interest was the MCC for each drug. Medians and interquartile ranges (IQRs) were used to describe the center and spread of the MCC for each medication, rather than means and standard deviations, because this measure followed a log_2_ distribution as a result of the serial two-fold dilutions. To quantify the relative potency of each medication, we determined the number of two-fold dilutions required to reach the MCC. The more dilutions required to reach a noncysticidal concentration, the higher the potency of the standard formulation of the medication. The number of two-fold dilutions required to reach the MCC was compared between drugs using the Kruskal-Wallis test, and permutation tests with 1000 replications each were performed for pairwise comparisons between drugs.^21^,^22^ The Bonferroni correction was applied to all *P* values to adjust for multiple comparisons. All statistical analyses were performed using *R* software version 4.0.2 (R Foundation for Statistical Computing). This study did not involve human subjects.

## Results

**Figure 1:**
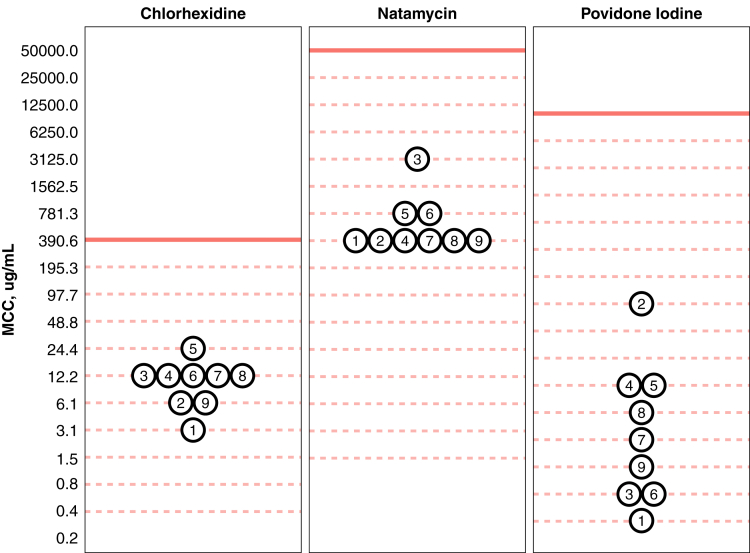
Dot plot demonstrating the minimum cysticidal concentrations (MCCs) of chlorhexidine, natamycin, and povidone iodine among 9 *Acanthamoeba* isolates. Note the y-axis scale has been log_2_-transformed. Solid red lines indicate the concentration of undiluted formulations of each agent (chlorhexidine 0.04%, natamycin 5%, and povidone iodine 1%) in micrograms per milliliter. All *Acanthamoeba* specimens demonstrated MCC levels of less than the full-strength concentrations of each antimicrobial. Dashed red lines indicate the concentrations of serial two-fold dilutions of each agent. Specimen identification numbers are plotted within each dot. The specimen with the highest MCC for povidone iodine (specimen 2) was not the same as the specimen with the highest MCC for natamycin (specimen 3) or chlorhexidine (specimen 5).

Minimum cysticidal concentrations for each antimicrobial among the 9 *Acanthamoeba* isolates are depicted in Figure 1. For all assays, the estimated MCC was lower than the undiluted formulation, that is, the drug concentration typically used in clinical practice. The MCCs of chlorhexidine ranged from 3.12 μg/ml to 25 μg/ml among the 9 *Acanthamoeba* isolates, with a median of 12.5 μg/ml (IQR, 6.25–12.5 μg/ml). A solution of 0.04% chlorhexidine could undergo a median of 5 (IQR, 5–6) two-fold dilutions before losing cysticidal activity. The range of MCCs for natamycin was 390.6 to 3125 μg/ml, with a median of 390.6 μg/ml (IQR, 390.6–781.2 μg/ml). Natamycin 5% could undergo a median of 7 (IQR, 6–7) two-fold dilutions before becoming noncysticidal. For povidone iodine, MCCs ranged from 0.3 to 78.1 μg/ml, with a median MCC of 2.4 μg/ml (IQR, 0.6–9.8 μg/ml). A 1% solution of povidone iodine could undergo a median of 12 (IQR, 10–14) two-fold dilutions before losing its cysticidal activity.

A statistically significant difference was found in the potency of the stock solution concentrations of these 3 medications (*P* < 0.001, Kruskal-Wallis test comparing number of two-fold dilutions before becoming noncystidal). Specifically, povidone iodine 1% could undergo 6.4 more two-fold dilutions than chlorhexidine 0.04% (*P* < 0.001, permutation test) and 5.3 more dilutions than natamycin 5% (*P* < 0.001, permutation test) before becoming noncysticidal. The mean number of dilutions required for natamycin 5% to reach the MCC was 1.1 higher than chlorhexidine 0.04%, although this difference was not statistically significant (*P* = 0.16, permutation test). The concentrations chosen for the stock solutions were based on those commonly used in clinical practice for *Acanthamoeba* keratitis. Concentrations of chlorhexidine as high as 0.2% (2000 μg/ml) have been described for other types of infectious keratitis, which would allow 7.7 two-fold dilutions before reaching the MCC, but still be less potent than the 1% povidone iodine solution (4.1 fewer dilutions; *P* < 0.001, permutation test).^10^ The specimen with a relatively higher MCC for natamycin (specimen 3) was not the same specimen that demonstrated a relatively higher MCC for povidone iodine (specimen 2; Fig 1).

## Discussion

In this study, we applied an established cysticidal assay method to determine and compare the relative potency of typical concentrations of 3 antimicrobial agents used clinically against *Acanthamoeba* cysts: povidone iodine 1%, chlorhexidine 0.04%, and natamycin 5%. We found that although some variability occurred in susceptibility among the different *Acanthamoeba* specimens tested, in every case, the minimum cysticidal concentration was less than the standard undiluted formulations of these 3 antimicrobials. This is generally consistent with prior studies evaluating the antiamebic activity of these 3 agents, which is notable given the significant variability and measurement noise inherent in cysticidal assays. We also determined that povidone iodine 1% had a statistically significantly higher potency than the other 2 agents (chlorhexidine 0.04% and natamycin 5%) as measured by the number of dilutions required to reach the minimum cysticidal concentration. Controlled clinical studies are required to determine whether these in vitro differences in potency translate to differential medication efficacy in vivo.

Povidone iodine is rapidly cytotoxic to prokaryotic cell membranes and has a broad spectrum of antimicrobial activity against bacteria, fungi, viruses, and protozoa, even at concentrations of 0.5% or less.^23^^,^^24^ It has the additional advantage of being inexpensive, chemically stable at room temperature, and widely available, which is particularly important given the burden of infectious keratitis in the developing world.^25^ Table 1 summarizes the results of prior studies examining the cysticidal effects of povidone iodine, natamycin, and chlorhexidine. Although several studies have evaluated the amoebicidal effects of combination solutions containing povidone iodine for contact lens decontamination, few have quantified the cysticidal activity of povidone iodine alone*.*^26^, ^27^, ^28^ One recent study from Japan demonstrated 100% cysticidal effect of povidone iodine 0.1% when tested on 56 *Acanthamoeba* strains (i.e., a cysticidal concentration of 1000 μg/ml or less), but performed no further dilutions to determine a precise MCC.^12^ The investigators of this study identified significant morphologic damage to cysts on transmission electron microscopy after exposure to povidone iodine. Similar electron microscopy findings were observed in a study of *Acanthamoeba* specimens from Thailand, which estimated an MCC of 400 μg/ml.^12^^,^^13^ A study from Italy measured an MCC between 250 and 500 μg/ml for povidone iodine, although in this study, povidone iodine had no cysticidal activity against 1 strain.^14^ In contrast, a study from Australia identified no in vitro cysticidal effect of povidone iodine at concentrations of 256 μg/ml in any of 19 *Acanthamoeba* specimens, although these were cysts collected from water sources and were not known to have caused keratitis.^15^ The median MCC of 2.4 μg/ml in our study is the lowest yet reported for povidone iodine. Variability in cysticidal activity across studies may be attributable to regional differences in susceptibility patterns or to differences in cysticidal assay methods.

**Table 1:** Summary of Published In Vitro *Acanthamoeba* Cysticidal Assay Results for Povidone Iodine, Natamycin, and Chlorhexidine

Antimicrobial Agent	Species Tested	No.	Specimen Source	Encystment Method∗	Growth Method†	Reported Cysticidal Activity	Reference No.
Povidone iodine	NR	9	Clinical	Time	Bacterized	Median MCC, 2.4 μg/ml (IQR, 0.6–9.8 μg/ml; range, 0.3–78.1 μg/ml)	‡
	*Castellanii*	56	Clinical	Time	Bacterized	MCC not reported, but 100% of strains were susceptible to povidone iodine 1% after 24-hr exposure	12
	NR	19	Water	NR	Bacterized	Median MCC, > 256 μg/ml	15
	NR	1	Clinical	Time	Bacterized	MCC, 400 μg/ml	13
	Multiple	6	Library	Starvation	Axenic	MCC, < 0.25%–> 10% (mean not reported)	14
	*Castellanii*	1	Library	Neff’s	Bacterized	2.8 log_10_ kill	26
	*Castellanii*	1	Library	NR	Bacterized	Only 1 concentration tested (0.4%), which was not cysticidal	27
Natamycin	NR	9	Clinical	Time	Bacterized	Median MCC, 390.6 μg/ml (IQR, 390.6–781.2 μg/ml; range, 390.6–3125 μg/ml)	‡
	*Castellanii*	56	Clinical	Time	Bacterized	MCC not reported, but 100% of strains were susceptible to natamycin 5% after 24-hr exposure	12
	Multiple	11	Clinical	Starvation	Axenic	Median MCC, 16 μg/ml (range, 2–128 μg/ml)	42
	NR	5	Clinical	Time	Bacterized	MCC_90_, 11.6 μg/ml	31
Chlorhexidine	NR	9	Clinical	Time	Bacterized	Median MCC, 12.5 μg/ml (IQR, 6.25–12.5 μg/ml; range, 3.12–25 μg/ml)	‡
	NR	13	Clinical	Time	Bacterized	Mean MCC 2.8 μg/ml (range, 0.49–15.6 μg/ml)	17
	*Castellanii*	56	Clinical	Time	Bacterized	MCC not reported, but 100% of strains were susceptible to chlorhexidine 0.02% after 24-hr exposure	12
	*Castellanii*	3	Library	Neff’s	Axenic	MCC not reported, but 100% of strains were susceptible to chlorhexidine 25 μg/ml (0.002%) after 30-min exposure	43
	*Castellanii*	15	Library, clinical	Time	Bacterized	MCC not reported, but 50% of strains were susceptible to chlorhexidine 0.02% after 72-hr exposure	44
	NR	19	Water	NR	Bacterized	Median MCC, 32 μg/ml	15
	NR	25	Clinical	Time	Bacterized	Mean MCC, < 5 μg/ml	16
	Multiple	6	Library	Time	Bacterized	MCC, 0.0125%–>0.1% (mean not reported)	14
	NR	19	Clinical	Time	Bacterized	Mean MCC, 32.8 μg/ml (range, 1.56–100 μg/ml)	18

IQR = interquartile range; MCC = minimum cysticidal concentration; NR = not reported.

∗ Neff’s method: use of specific growth media; starvation method: sudden removal of nutrients; time method: leaving organisms in growth media and allowing to encyst; see Shoff and Eydelman.^19^

† “Axenic” indicates a growth medium without bacteria; “bacterized” indicates the use of bacteria as a food source.

‡ Present study.

The relationship between the concentration of povidone iodine and its antimicrobial efficacy is complex. Paradoxically, the amount of free iodine and thus the potential microbicidal effect increases as povidone iodine concentration decreases, peaking when the concentration is between 0.1% and 1%.^29^ Conversely, Ferguson et al^30^ showed that povidone iodine 5% was more effective than povidone iodine 1% in reducing bacterial colonization of the conjunctiva before surgery. Toxicity also must be considered when determining an optimal therapeutic concentration; povidone iodine causes increasing damage to corneal fibroblasts as concentration increases beyond 100 μg/ml.^23^ Our results indicate that effective concentrations may be achievable without significant toxicity considering that the MCC of povidone iodine was less than 100 μg/ml for every *Acanthamoeba* specimen tested in this study.

Natamycin is an antifungal agent that affects membrane permeability and has been shown to be cysticidal in prior studies, inducing significant morphologic changes on transmission electron microscopy.^12^^,^^31^ However, its penetration into the corneal stroma is limited when the epithelium is intact.^32^ Several case reports have been published describing variable clinical effect in *Acanthamoeba* keratitis, but no randomized trials have been performed to date.^33^, ^34^, ^35^ Chlorhexidine is a cationic antiseptic agent of the biguanide family with a broad spectrum of activity via destruction of microbial cell walls and plasma membranes.^36^ The biguanides, particularly polyhexamethylene biguanide and chlorhexidine, have demonstrated the most consistent cysticidal activity in vitro of all antiacanthamebal agents.^10^^,^^12^^,^^15^, ^16^, ^17^, ^18^ The MCC of chlorhexidine in these reports ranges from 1.5 to 100 μg/ml, which is consistent with our median result of 12.5 μg/ml. Of note, prior studies have found chlorhexidine to cause less observable morphologic damage on transmission electron microscopy and to have a time-dependent effect, unlike natamycin and povidone iodine.^12^

In this study, povidone iodine 1% was statistically significantly more potent than natamycin 5% or chlorhexidine 0.04%, on average requiring approximately 12 two-fold dilutions to reach noncysticidal concentrations. Although all 3 medications were cysticidal in every case at higher concentrations, the measured differences in relative potency theoretically could have practical clinical importance considering that not all molecules of a topically applied compound reach the target microbes. Topical ophthalmic medications are diluted rapidly by tears, and penetration into the corneal stroma is variable depending on the hydrophilicity of the compound and the integrity of the corneal epithelium.^37^ The only other published study to compare directly the in vitro cysticidal activity of povidone iodine, natamycin, and chlorhexidine similarly found that povidone iodine 1%, natamycin 5%, and chlorhexidine 0.02% were cysticidal in all 56 *Acanthamoeba* strains tested after 24 hours of exposure to the antimicrobial agent.^12^ In that study, povidone iodine and natamycin were also 100% cysticidal after undergoing a single 10-fold dilution, whereas chlorhexidine 0.002% was cysticidal in only 45% of strains. However, this was based on only a single dilution, and MCC was not reported.

The cysticidal effect of the 3 agents studied demonstrated moderate variability among different *Acanthamoeba* specimens, which is consistent with prior studies. However, all MCC levels were well below the undiluted formulations of these antimicrobial agents, so the practical significance of this variability is unclear. In our experience, much of this variability is likely the result of noisy antiamebic susceptibility assays, because we often observe variability in assays run on the same organism. Other components of variability could include differences in *Acanthamoeba* species and genomic profiles. Variable susceptibility patterns have been observed in *Acanthamoeba* cysts with the same T4 genotype, so genomic differences likely do not completely explain observed variability in antimicrobial sensitivity.^12^ *Acanthamoeba* species are also known to have a diverse array of endosymbionts, which may play a role in differential susceptibility and clinical response to therapy.^38^ Gene and protein expression studies in combination with in vitro susceptibility testing are required to determine the underlying causes of this variability.

We acknowledge several limitations of this study. First, in vitro results do not predict in vivo outcomes. Discordance between laboratory susceptibility testing and clinical response to therapy in *Acanthamoeba* keratitis has been described repeatedly in the literature.^15^^,^^17^^,^^39^ Second, we examined a small number of specimens from a single center in the United States; these results may not be geographically generalizable and should be interpreted accordingly. Third, we used an established assay method described previously in the literature, but determination of the MCC of antimicrobial agents may differ when alternative assays are used.^16^^,^^17^^,^^20^ This should be considered when comparing results across different studies. Fourth, differences in storage conditions and encystment or excystment methods can impact the viability and resistance patterns of *Acanthamoeba* specimens, which should be considered when comparing the results of this work with those of prior studies.^19^^,^^40^^,^^41^ However, prior research has suggested that the main driver of variability in resistance patterns is the growth method, with axenic methods (i.e., nutrient media without bacteria) resulting in more susceptible acanthamoeba and bacterized growth methods such as those used in the present study resulting in more resistant bacteria.^19^ Thus, studies like this one that use bacterized growth methods may provide more conservative estimates of susceptibility patterns for acanthamoeba. Fifth, we did not examine the species or genotype of *Acanthamoeba* specimens in this study, both of which likely play a role in susceptibility patterns to various antimicrobials. Finally, time-dependent and concentration-dependent mechanisms may play a role for different antimicrobials in the treatment of *Acanthamoeba* keratitis.^12^ Because of a fixed exposure time to each medication, we could not evaluate this possibility in our study. However, the clinical impact of these effects may not be significant considering the typical frequency of instillation and duration of medical therapy in the management of *Acanthamoeba* keratitis.

In conclusion, povidone iodine, chlorhexidine, and natamycin demonstrated complete in vitro cysticidal effect against all *Acanthamoeba* specimens at concentrations well below standard formulations. Povidone iodine demonstrated the highest potency of the 3 agents and may have a role in medical therapy for *Acanthamoeba* keratitis. These results inform the design and implementation of a randomized clinical trial assessing the clinical efficacy of povidone iodine in combination with chlorhexidine to address this question.
